# Chemical Composition and In Vitro Antimicrobial Efficacy of Sixteen Essential Oils against *Escherichia coli* and *Aspergillus fumigatus* Isolated from Poultry

**DOI:** 10.3390/vetsci5030062

**Published:** 2018-06-25

**Authors:** Valentina Virginia Ebani, Basma Najar, Fabrizio Bertelloni, Luisa Pistelli, Francesca Mancianti, Simona Nardoni

**Affiliations:** 1 Department of Veterinary Science, University of Pisa, viale delle Piagge 2, 56124 Pisa, Italy; fabrizio.bertelloni@vet.unipi.it (F.B.); francesca.mancianti@unipi.it (F.M.); simona.nardoni@unipi.it (S.N.); 2 Centro Interdipartimentale di Ricerca “Nutraceutica e Alimentazione per la Salute”, University of Pisa, via del Borghetto 80, 56124 Pisa, Italy; luisa.pistelli@unipi.it; 3 Department of Pharmacy, University of Pisa, via Bonanno 6, 56126 Pisa, Italy; basmanajar@hotmail.fr

**Keywords:** antimicrobial activity, *Escherichia coli*, *Aspergillus fumigatus*, poultry

## Abstract

*Escherichia coli* and *Aspergillus fumigatus* are two pathogens largely present among poultry. They can cause mild or severe forms of disease, and are associated with significant economic losses. The aim of the present study was to investigate the chemical composition and the in vitro antimicrobial activity of sixteen essential oils (EOs) and five mixtures against *E. coli* and *A. fumigatus* strains previously isolated from poultry. The study was performed with the following EOs: *Aloysia* *tryphilla*, *Boswellia* *sacra*, *Cinnamomum zeylanicum*, *Citrus aurantium*, *Citrus bergamia*, *Citrus limon*, *Citrus reticulata*, *Cymbopogon citratus*, *Eucalyptus globulus*, *Lavandula hybrida*, *Litsea cubeba*, *Ocimum basilicum*, *Melaleuca alternifolia*, *Mentha piperita*, *Pelargonium* *graveolens*, and *Syzygium aromaticum*. Moreover, the following mixtures were also tested: *L. cubeba* and *C. citratus* (M1), *L. cubeba* and *A. triphylla* (M2), *A. triphylla* and *C. citratus* (M3), *A. triphylla*, *C.* *citratus* and *L. cubeba* (M4), *S. aromaticum* and *C. zeylanicum* (M5). One hundred and ninety-one compounds were identified in the tested EOs and mixtures. MIC determination found good anti-*E. coli* activity with *C. zeylanicum* (2.52 mg/mL), *C. citratus* (1.118 mg/mL), *L. cubeba* (1.106 mg/mL), *M. piperita* (1.14 mg/mL) and *S. aromaticum* (1.318 mg/mL) EOs. Among the mixtures, M5 showed the best result with a MIC value of 2.578 mg/mL. The best antimycotic activity was showed by *A. triphylla* (0.855 mg/mL), followed by *C. citratus* (0.895 mg/mL), while *C. aurantium*, *M. piperita*, *B. sacra* and *P. graveolens* did not yield any antifungal effect at the highest dilution. The mixtures exhibited no antifungal activity at all. This study shows promising results in order to use EOs in the environment for disinfection purposes in poultry farms and/or in hatcheries.

## 1. Introduction

*Escherichia coli* and *Aspergillus fumigatus* are two pathogen agents which are largely present in poultry flocks and can cause able to cause mild or severe forms of disease associated with economic losses. Improper hygiene conditions and animal immune system deficiencies would influence the severity of the infection outcome. Environmental stressors such as poor ventilation, warm temperature, excessive ammonia and moisture, degraded litters, long-term storage of feed may increase the concentration of fungal spores and bacteria in the farm environment [1,2,3].

Avian colibacillosis is a systemic disease of poultry caused by avian pathogenic *Escherichia coli* (APEC) strains. This is an enteric bacterium, which infects the animals by an oral-fecal cycle, even though it often affects birds by inhalation of contaminated dust.

The infection usually starts with septicemia followed by localized inflammation in multiple organs or sudden death [4]. Colibacillosis is characterized by variable lesions, among which airsacculitis and polyserositis are the most frequent. Moreover, fecal contamination of the eggs may cause *E. coli* penetration through the shell. This may result both in yolk sac bacterial infection and in spreading to other chickens during hatching, with high mortality rates. Economic losses may be also due to slaughter waste related to necrotic cellulitis [5].

Avian aspergillosis is a fungal disease caused by members of the genus *Aspergillus*, mainly *Aspergillus fumigatus*. The most common forms of avian aspergillosis are represented by lung infections in poultry and other different bird species. Fungal conidia are inhaled and penetrate the respiratory system colonizing the air sacs, and large inoculum of conidia is considered a main causative factor [6]. Infection can occur in hatchery also, due to infected eggs that accidentally open during incubation or hatching, releasing large number of spores in the environment. Furthermore, aspergilli are reported as responsible for brooder pneumonia, when spores penetrate the egg shell. Chicks develop acute respiratory disease that can be lethal in the first 1–3 weeks of age. Chronic aspergillosis is sporadic and usually affects older birds [7].

This mycosis is responsible for economic losses linked to mortality, reduced feed conversion ratio and to carcass condemnation at slaughter inspection due to airsacculitis [8].

Use of antibiotics is necessary to resolve bacterial infections, but their employment has certain disadvantages including: antibiotic residues in meat and eggs and the selection of multi-drug resistant pathogens, that can spread within the farm population such as among other animals.

Conversely antimycotic drugs are not allowed in animals intended for human consumption, and the treatment of affected poultry would be too expensive, anyway. Therefore, the prevention of both fungal and bacterial infections appears to be fundamental [3].

The use of compounds derived from plants, such as essential oils (EOs), may be a good alternative to prevent the spreading of microorganisms in the poultry farm environments, mainly hatcheries and cages. However, data about the effectiveness of EOs in improving hygienic conditions are very scant [9]. Data about the effectiveness of air-dispersed EOs in reducing bacterial and fungal burden in nosocomial environment, suggest a possible application of these natural substances vapours to control undesired agents.

The aim of the present study was to investigate the chemical composition and the in vitro antimicrobial activity of sixteen EOs derived from different botanical species, alone or in mixture, against *E. coli* and *A. fumigatus* strains previously isolated from poultry. EOs were chosen on the basis of their commercial availability, odor characters and no toxicity in view of a possible environmental use.

## 2. Materials and Methods

### 2.1. Essential Oils

The antimicrobial activity of the following sixteen EOs, kindly provided by the producer (FLORA^®^, Pisa, Italy), was investigated: lemon verbena (*Aloysia tryphilla* (L’Hèr.) Britton), incense (*Boswellia sacra* Flueck.), cinnamon (*Cinnamomum zeylanicum* J. Presl), bitter orange (*Citrus aurantium* L.), bergamot (*Citrus bergamia* Risso & Poit.), lemon (*Citrus limon* (L.) Osbeck), mandarin (*Citrus reticulata* Blanco), lemon grass (*Cymbopogon citratus* (DC.) Stapf), eucalyptus (*Eucalyptus globulus* Labill.), lavender (*Lavandula hybrida*), litsea (*Litsea cubeba* (Lour.) Pers.), basil (*Ocimum basilicum* L.), tea tree (*Melaleuca alternifolia*), peppermint (*Mentha piperita*), geranium (*Pelargonium graveolens* L’Hèr.), clove (*Syzygium aromaticum* (L.) Merr. & L.M. Perry).

After preliminary results about the efficacy of each EO, mixtures have been made with the most active ones. Mixtures were prepared with 1:1 proportion (*w*/*w*) of *L. cubeba* and *C. citratus* (M1), *L. cubeba* and *A. triphylla* (M2), *A. triphylla* and *C. citratus* (M3), as well as for *S. aromaticum* and *C. zeylanicum* (M5) together with a mixture of three EOs (M4) obtained with *A. triphylla*, *C. citratus* and *L. cubeba* (1:1:1). All EOs and mixtures were maintained at 4 °C in dark glass vials until their use. Then they were microbiologically analyzed for quality control before antibacterial and antimycotic activity tests. At this purpose, a loopful of each EO was streaked onto a blood agar plate and the plates were incubated at 37 °C for 48 h.

Fractional Inhibitory Concentration Index (FICI) was calculated as reported by Doern [10] to evaluate the possible synergistic effect in the mixtures. The FICI was interpreted as: a synergistic effect when ≤0.5; an additive effect when >0.5–1; indifferent effect when 1–4 and an antagonistic effect when >4.

### 2.2. Essential Oils Analysis

All the selected EOs and mixtures were analyzed using Gas Cromatography-Mass Spectrometry (GC-MS) according to the method cited in a previous paper, as well as for the identification of the different compounds [11].

### 2.3. Statistical Analysis

Regarding the huge variety of the selected EOs, obtained from plants belonging to different orders, families and species, a statistical analysis approach was applied to evaluate the results of their composition. Hierarchical Cluster Analysis (HCA) was performed using Ward’s method and squared Euclidian distances for the measure of similarity. Moreover, the Principal Component Analysis (PCA) was done by ‘Past 3 software package’ version 3.15.

### 2.4. Antibacterial Activity

#### 2.4.1. Bacterial Strain

An *Escherichia coli* strain, previously isolated in a case of poultry colibacillosis, was employed in the study. The isolate was typed using the API20E System (BioMérieux, Marcy l’Etoile, France) and stored in glycerol broth at −80 °C until used.

#### 2.4.2. Agar Disc Diffusion Method

Antibacterial activity of each EO and mixture was tested by Kirby-Bauer agar disc diffusion method following the procedures previously described [12]. A 1:10 dilution in dimethyl sulfoxide (DMSO, Oxoid Ltd., Basingstoke, Hampshire, UK) of each EO and mixture was assayed. All tests were performed in triplicate.

The in vitro sensitivity of *E. coli* strain to amoxycillin-clavulanic acid (30 μg) (Oxoid) was evaluated by Kirby-Bauer method and the results were interpreted as indicated by Clinical and Laboratory Standards Institute [13].

#### 2.4.3. Minimum Inhibitory Concentration

Minimum inhibitory concentration (MIC) was determined for all EOs and mixtures with the broth microdilution method, starting from a dilution of 10% (*v*/*v*) and following the guidelines of CLSI [14] and a protocol previously reported [12]. All tests were performed in triplicate. The MIC value was determined as the lowest concentration, expressed in mg/mL, of each EO and mixture at which bacteria show no visible growth.

### 2.5. Antimycotic Activity

#### 2.5.1. Fungal Strain

An avian clinical isolate of *A. fumigatus* was employed for testing. The mold was maintained onto malt extract agar (MEA), and identification was accomplished on the basis of macroscopic and microscopic features on both MEA and Czapeck agar, following the keys provided by Raper and Fennel [15].

#### 2.5.2. Minimum Inhibitory Concentrations

MICs were determined by a microdilution test carried out as reported elsewhere [12], starting from a dilution of 5% (*v*/*v*), following the methods described by CLSI [16] for molds. All tests were performed in triplicate and positive controls using a conventional antimycotic drug (voriconazole) were also performed by microdilution test.

## 3. Results

### 3.1. Essential Oil and Mixture Composition

**Figure 1:**
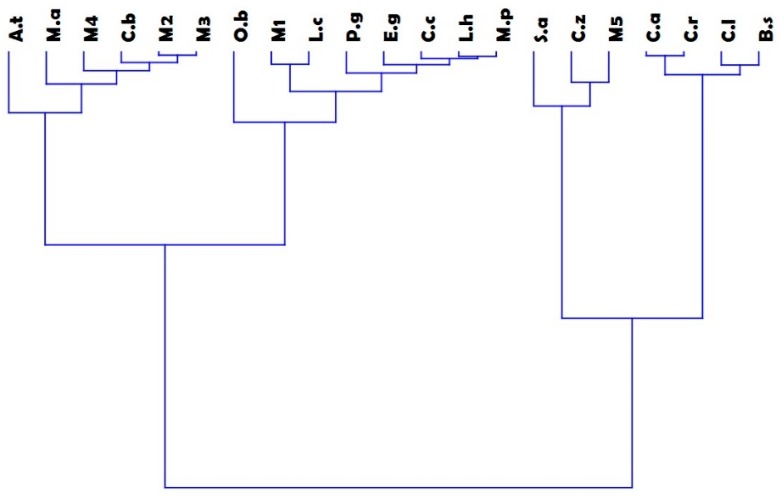
Dendrogram of the Hierarchical Cluster Analysis (HCA) of both essential oils and mixtures. Legend: *L. h*: *Lavandula hybrida*, *M. p*: *Mentha piperita*, *O. b*: *Ocimum basilicum*, *A. t*: *Aloysia triphilla*, *C. c*: Legend: *L. h*: *Lavandula hybrida*, *M. p*: *Mentha piperita*, *O. b*: *Ocimum basilicum*, *A. t*: *Aloysia triphilla*, *C. c*: *Cymbopogon citratus*, *C. z*: *Cinnamomum zeylanicum*, *L. c*: *Litsea cubeba*, *P. g*: *Pelargonium graveolens*, *E. g*: *Eucalyptus globulus*, *M. a*: *Melaleuca alternifolia*, *S. a*: *Syzygium aromaticum*, *B. s: Boswellia sacra*, *C. a*: *Citrus aurantium*, *C. b*: *Citrus bergamia*, *C. l*: *Citrus limon*, *C. r*: *Citrus reticulata*, M1: *Litsea cubeba–Cymbopogon citratus*, M2: *Aloysia triphylla–Listea cubeba*, M3: *Aloysia triphylla–Cymbopogon citratus*, M4: *Aloysia triphylla–Litsea cubeba–Cymbopogon citratus*, M5: *Cinnamomum zeylanicum–Syzygium aromaticum*.

One hundred and ninety-one compounds were detected in the tested EOs and mixtures (Table 1). Table 2 shows the percentages of the main class of constituents in each EO tested. HCA showed two main groups (A and B), each of them also divided in two subgroups (Figure 1). The first subgroup (A1) included the following species: *A. triphylla*, *M. alternifolia*, *C. bergamia* and mixtures M2, M3 and M4. It is important to underline that *A. triphylla* EO was one of EOs present in four mixtures. *O. basilicum*, *L. cubeba*, *P. graveolens*, *E. globulus*, *C. citratus*, *L. hybrida*, *M. piperita* together with M1 mixture were inserted in subgroup (A2). M1 mixture consisted of two EOs categorized in the same subgroup (*L. cubeba* and *C. citratus*).

The EOs obtained from the remaining botanical species and mixture M5 belonged to the second group (B), which was divided in two subgroups: (B1) with *S. aromaticum*, *C. zeylanicum* and mixture M5; while the subgroup (B2) gathered four out of the five EOs belonging to the Sapindales Order.

**Figure 2:**
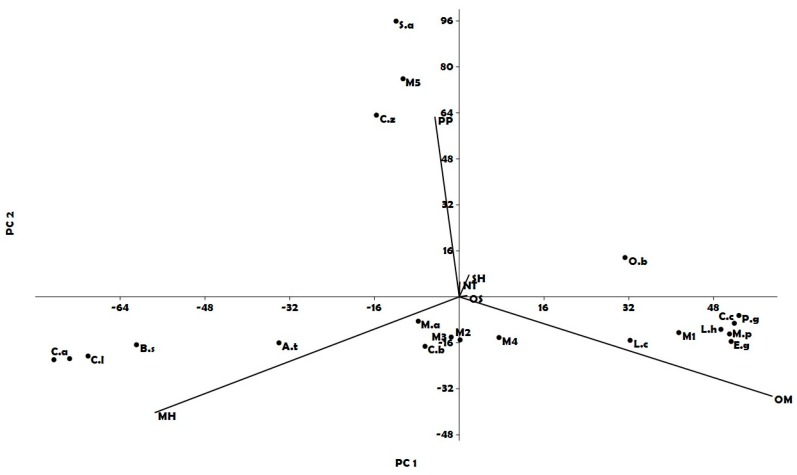
The compound analysis plot (PCA) of the main classes of compounds in the different essential oils and mixtures analyzed. Legend: *L. h*: *Lavandula hybrida*, *M. p*: *Mentha piperita*, *O. b*: *Ocimum basilicum*, *A. t*: *Aloysia triphilla*, *C. c*: Legend: *L. h*: *Lavandula hybrida*, *M. p*: *Mentha piperita*, *O. b*: *Ocimum basilicum*, *A. t*: *Aloysia triphilla*, *C. c*: *Cymbopogon citratus*, *C. z*: *Cinnamomum zeylanicum*, *L. c*: *Litsea cubeba*, *P. g*: *Pelargonium graveolens*, *E. g*: *Eucalyptus globulus*, *M. a*: *Melaleuca alternifolia*, *S. a*: *Syzygium aromaticum*, *B. s: Boswellia sacra*, *C. a*: *Citrus aurantium*, *C. b*: *Citrus bergamia*, *C. l*: *Citrus limon*, *C. r*: *Citrus reticulata*, M1: *Litsea cubeba–Cymbopogon citratus*, M2: *Aloysia triphylla–Listea cubeba*, M3: *Aloysia triphylla–Cymbopogon citratus*, M4: *Aloysia triphylla–Litsea cubeba–Cymbopogon citratus*, M5: *Cinnamomum zeylanicum–Syzygium aromaticum*.

HCA analysis gave only limited information; therefore, a further statistical analysis was important to better understand this partition. PCA plot, where the two first axis explain for more than 98.3% of variability, grouped the EOs species among 3 main quadrants: two lower quadrants characterised by high amount of monoterpene hydrocarbons (MH) on the left and by an important percentage of oxygenated monoterpenes (OM) on the right. The upper left quadrant included two EOs (clove and cinnamon) and their mixture M5, in which the amount of phenylpropanoids (PP) was more than 60% (Figure 2).

In detail, the EOs present in the lower left quadrant all belonged to Sapindales order. Among these species, *C. reticulata*, *C. aurantium*, *C. limon* and *C. bergamia* belonged to the same Rutaceae family. The first three species showed the highest percentage of MH (99.7%, 97.4% and 94.3%, respectively) while *C. bergamia* evidenced an equivalent amount between MH and OM (49.0% and 48.5%, respectively). Bergamot was therefore positioned in the middle between EOs characterised by an important percentage of MH and OM. *B. sacra*, which belongs to the Burseraceae family in the Sapindales order, showed a good percentage of MH (84.3%) and was near the other *Citrus* spp. Regarding Lamiales order, only *L. hybrida* and *M. piperita* were present in the lower right quadrant of PCA, due to the high amount of OM (85.0% and 87.8%, respectively), together with *E. globulus*, *C. citratus* and *P. graveolens* characterized by a similar high percentage of the same constituents (OM: 90.5, 86.3 and 83.4%, respectively). The other two EOs from species belonging to Lamiaceae, showed some distance from each other: in fact, *O. basilicum* presented a lower percentage of OM (56.1%) but also the highest amount of sesquiterpene hydrocarbons (SH) (20.0%) and was positioned in the upper right quadrant, while lemon verbena, which belongs to the Verbenaceae family (Lamiales order), was on the opposite side due to its relevant amount of MH (66.0%).

*L. cubeba* from Lauraceae family showed high percentage of OM (75.7%) and a good amount of MH (21.3%). This aromatic profile differed from the others in the same quadrant.

The position of the mixtures in PCA analysis followed the EOs that took part in their composition. In fact, M1 was located in the right lower quadrant as well as *L. cubeba* and *C. citratus* with a content of 80.0% of MH. Moreover, M2 was found in the middle between *L. cubeba* and *A. triphylla*, while M3 was positioned between *A. triphylla* and *C. citratus*. M4, which was obtained mixing three EOs (*A. triphylla*, *C. citratus* and *L. cubeba*, 1:1:1) was shifted on the right quadrant due to the high percentage of OM (56.0%).

### 3.2. Antibacterial Activity

Agar disc diffusion method revealed growth inhibition zones with the following EOs: *C. zeylanicum*, *C. citratus*, *L. cubeba*, *M. piperita*, *O. basilicum*, *P. graveolens* and *S. aromaticum*. Antibacterial activity was revealed also testing the five mixtures. No inhibition zone was observed with the remaining oils and the negative control. *E. coli* tested against amoxycillin-clavulanic acid resulted sensitive with an inhibition zone of 20 mm and a MIC value of 0.008/0.004 mg/mL.

MIC determination found good anti-*E. coli* activity with *C. zeylanicum* (2.52 mg/mL), *C. citratus* (1.118 mg/mL), *L. cubeba* (1.106 mg/mL), *M. piperita* (1.14 mg/mL) and *S. aromaticum* (1.318 mg/mL) EOs, whereas *O. basilicum* and *P. graveolens* resulted effective to *E. coli*, but with higher MIC values (9.15 mg/mL and 17.8 mg/mL) (Table 3).

Among the tested mixtures, M5 showed the best result with a MIC value of 2.578 mg/mL. FICI calculation highlighted an indifferent effect between the two EOs present in M5. The remaining mixtures did not show good antibacterial activity, as also established by FICI that revealed antagonistic effect between the mixtures components.

### 3.3. Antimycotic Activity

The selected EOs showed different patterns of efficacy. *A. triphylla* appeared to be the most effective (0.855 mg/mL) followed by *C. citratus* (0.895 mg/mL), while *C. aurantium*, *M. piperita*, *B. sacra* and *P. graveolens* did not yield any antifungal effect at the highest dilution. The fungal isolate resulted sensitive to 1 mg/L of voriconazole. The mixtures exhibited no antifungal activity at all, when tested undiluted, as indicated by FICI that showed antagonistic effects among the mixtures components.

## 4. Discussion

The present study takes into account the efficacy of a conspicuous number of EOs against two phylogenetically distant organisms, both involved in impairing poultry health and breeding hygiene. Wild type bacterial and fungal strains were selected to better resemble field conditions.

Our results showed that selected EOs exhibited different antimicrobial activity against the tested pathogen agents.

Cinnamon and clove EOs evidenced good antibacterial activity, when used alone or in combination (M5). This action may be related to the main compounds present in these EOs, eugenol (77.9%) and its acetate form (12.2%), as suggested in other studies, too [17].

Zhang et al. [18] observed that cinnamon EO induces damage on permeability and integrity of membrane with consequent loss of inner cell materials. Similar effect against *E. coli* was found with clove EO by Rhayour and coworkers [19] who observed a damage as holes in both cell wall and membrane.

*L. cubeba* EO has a relevant anti-*E. coli* activity, as previously observed by Li et al. [20], who documented holes and gaps on outer and inner membranes of *E. coli* cells treated with this EO, mainly attributed to the presence of aldehydes as geranial (36.4%) and neral (32.5%).

During this investigation *M. piperita* EO showed high activity against *E. coli*. These results are in agreement with Goudjil et al. [21], who tested *M. piperita* EO against some Gram positive and Gram negative bacteria and found the highest antimicrobial activity versus *E. coli*. Other authors verified the antimicrobial properties of peppermint EO and attributed the activity to the major components menthol and its oxidative compound menthone [22,23].

*C. citratus* revealed a good activity against *E. coli*, as also observed by other researchers who related this activity to the main components geranial and neral [24].

EOs from *O. basilicum* and *P. graveolens* showed moderate anti-bacterial activity corroborating other studies, which found higher effectiveness against Gram positive than Gram negative bacteria [25]. However, the antimicrobial property of basil EO was reported in other studies, in which a good activity against *E. coli* related to a significant amount of linalool was evidenced [26].

*A. triphylla*, *C. citratus* and *L. cubeba* EOs appeared to be the most active against *A. fumigatus*. These compounds would be of interest in poultry breeding, due to their low toxicity [27,28,29,30]. Furthermore, data available on the long lasting persistence of *C. citratus* EO [31] would suggest its feasibility, when used as disinfectant.

*A. triphylla* EO showed an interesting antifungal activity. Its composition is characterized by a 24% of sabinene, 36.7% of limonene and 12% of citronellal. The antifungal activity is probably due to sabinene, which appeared effective against *A. fumigatus* [32], as well as to citronellal [33]. Moreover, the present data are in full agreement with Correa-Royero et al. [34], who reported effectiveness against *A. fumigatus* at 99.2 µg/mL. *A. triphylla* was successfully assayed against *Candida* spp. both as EO [35,36] and as ethanolic extract [37], while it was not effective against *Trichoderma viride* [36].

In the present study, this EO was active at 0.5% concentration and such dilution appears to be safe for handling, following Tisserand and Young [27], who also recommend a maximum concentration of 0.9% for dermal application, to avoid phototoxic effects.

Lemongrass EO, with its high amount of both citral isomers, appeared to be another promising antifungal phytocomplex. This EO in fact is composed by a mixture of *cis* and *trans*-isomers of 3,7-dimethyl-2,6-octadiene-1-al (geranial and neral) with a percentage ranging between 38.4% and 35.2%, respectively.

Our result has been corroborated by Inouye et al. [38], who reported irreversible alterations in an in vitro model on apical growth of *A. fumigatus* elicited by *C. citratus* EO. Lemon, lavender and tea tree EOs induced a moderate and reversible inhibition, while cinnamon allowed a partial hyphal regrowth. Lemongrass exerted an antifungal activity against phytopathogenic fungi also at concentration up to 500 ppm [39].

*L. cubeba* EO showed neral and geranial content very similar to *C. citratus*, even if their MIC values were quite divergent (1.770 and 0.895, respectively), suggesting a different biological activity of phytocomplex.

## 5. Conclusions

This investigation shows promising results in order to use EOs for disinfection purposes in poultry farms and/or in hatcheries environment. The mixture with *C. zeylanimcum* and *S. aromaticum* could be a useful alternative treatment against *E. coli*. Moreover, *C. citratus* used alone could be employed to fight both *E. coli* and *A. fumigatus*, while *A. triphylla* EO would be used to enhance the control of spores’ burden, aiming to reduce the risk of infection for both animals and workers.

## Figures and Tables

**Table 1:** Chemical composition of tested EOs.

Clade			Asterids					Magnoliids		Rosids									Mixture (1:1)				
Order			Lamiales				Poales	Laurales			Myrtales			Sapindales									
Family			Lamiaceae					Lauraceae			Myrtaceae				Rutaceae								
			Relative Percentage (%) ^b^																				
Chemical Component		LRI ^a^	*L. h*	*M. p*	*O. b*	*A. t*	*C. c*	*C. z*	*L. c*	*P. g*	*E. g*	*M. a*	*S. a*	*B. s*	*C. a*	*C. b*	*C. l*	*C. r*	M1	M2	M3	M4	M5
α-Thujene	MH	930				0.2	0.1	0.3				0.9		54.2		0.3	0.4	0.5		0.1	0.2	0.2	
α-Pinene	MH	939		0.8	0.2						2.0	2.9		6.2					0.8	1.2	0.8	1.3	0.2
Thuja-2,4(10)-diene	MH	960												7.3									
Sabinene	MH	975	0.1	1.8	0.2	24.0		0.1	1.0			0.9		0.4	0.3	1.1	2.3	1.5	0.4	13.7	16.8	11.9	
β-Pinene	MH	979	0.4		0.5			0.5	1.2					1.1	0.1	5.4	11.9		0.6				
α-Phellandrene	MH	1003						2.1				0.5		3.7									0.3
α-Terpinene	MH	1017		0.2		0.2		1.0				9.1		5.1			0.2	0.4		0.2	0.1		0.1
*p*-Cymene	MH	1025		0.4		0.4		3.0	0.2		67.7	3.6				0.1	0.2	1.8	0.1	0.3	0.2		0.5
*o*-Cymene	MH	1026												3.3									
Limonene	MH	1029		3.0	0.3	36.7	2.0		16.3			3.0		0.4	94.7	33.2	65.7	72.1	9.1	24.6	21.7	20.2	
β-Phellandrene	MH	1030						5.9															1.1
1,8-Cineole	OM	1031	7.7	5.0	5.9		0.3		2.3		89.8	4.0											
γ-Terpinene	MH	1060	0.1	0.3		0.3			0.1			16.9		0.1		6.4	9.3	19.2		0.3	0.2	0.2	
Terpinolene	MH	1089	0.5	0.1	0.1	0.1		0.3				3.9		0.4		0.2	0.5	1.1		0.1	0.1		
Linalool	OM	1097	31.5	0.4	46.0	3.0	1.5	6.3	1.5	3.9				0.2	0.4	14.2			2.1	2.5	2.3	2.2	1.5
Camphor	OM	1146	7.3		0.8														0.4			0.3	
Menthone	OM	1153		26.6						1.1													
Citronellal	OM	1153				12.0	0.5		0.9										1.6	7.0	5.6	4.4	
*iso*-Menthone	OM	1163								3.5													
Menthofuran	OM	1164		12.5																			
Menthol	OM	1172		32.4																			
4-Terpineol	OM	1177	4.0		0.3	0.7		0.3	0.1			30.2							0.2	0.6	0.6	0.5	0.1
α-Terpineol	OM	1189	2.1	0.3	0.8	0.4		0.8	0.5	0.3		4.4				0.2	0.3		0.8	0.6	0.3	0.5	0.3
Citronellol	OM	1226				1.9				44.5									0.8	1.9	2.0	1.6	
Neral	OM	1238				0.7	35.2		32.5	0.2						0.4	0.7		32.0	17.2	14.7	18.6	
Geraniol	OM	1253			0.2		4.4		0.5	13.7									0.2	0.2	2.5	2.2	
Linalyl acetate	OM	1257	26.8												1.4	31.7							
Geranial	OM	1267				1.2	38.4		36.4	0.7						0.4	1.2		31.7	16.9	14.0	19.5	
*(E)*-Cinnamaldehyde	NT	1270						56.4															18.5
Citronellyl formate	OM	1274								7.3													
Menthyl acetate	OM	1295		6.1																			
Eugenol	pp	1359			11.5			3,0					77.9										51.7
β-Caryophyllene	SH	1419	2.2	2.8	0.3	1.3	2.3	10.3	0.8	0.7		0.8	8.9		0.2	0.7	0.4	0.1	1.6	1.2	1.6	1.5	7.6
Germacrene D	SH	1485	0.8	0.7	3.5	0.7	0.2			0.2				0.9						0.3	0.4	0.2	
Eugenyl acetate	PP	1523											12.2										12.7
δ-Cadinene	SH	1523		0.2	0.3		0.3	0.2		0.7		3.1	0.2						0.2		0.2	0.2	0.9
τ-Cadinol	OS	1640	0.2		5.8	0.1														0.1			
Unknown				0.2	0.8	0.5	0.4	0.3	0.6	0.7		1.8		5.1				0.2	1.8	1.5	1.8	1.3	0.1
**Total Identified**			100.0	99.8	99.2	99.5	99.6	99.7	99.4	99.3	100.0	98.2	100.0	94.9	100.0	100.0	100.0	99.8	98.2	98.5	98.2	98.7	99.9

^a^ Linear Retention Index, ^b^ compounds with percentage less than 3% in at least one of the EOs are not inserted in the table. Legend: *L. h*: *Lavandula hybrida*, *M. p*: *Mentha piperita*, *O. b*: *Ocimum basilicum*, *A. t*: *Aloysia triphilla*, *C. c*: *Cymbopogon citratus*, *C. z*: *Cinnamomum zeylanicum*, *L. c*: *Litsea cubeba*, *P. g*: *Pelargonium graveolens*, *E. g*: *Eucalyptus globulus*, *M. a*: *Melaleuca alternifolia*, *S. a*: *Syzygium aromaticum*, *B. s: Boswellia sacra*, *C. a*: *Citrus aurantium*, *C. b*: *Citrus bergamia*, *C. l*: *Citrus limon*, *C. r*: *Citrus reticulata*,—M1: *Litsea cubeba–Cymbopogon citratus*, M2: *Aloysia triphylla–Listea cubeba*, M3: *Aloysia triphylla–Cymbopogon citratus*, M4: *Aloysia triphylla–Litsea cubeba–Cymbopogon citratus*, M5: *Cinnamomum zeylanicum–Syzygium aromaticum*; MH: Monoterpene Hydrocarbons, OM: Oxygenated Monoterpenes, SH: Sesquiterpene Hydrocarbons, OS: Oxygenated Sesquiterpenes, PP: Phenylpropanoides, NT: Non-Terpenes.

**Table 2:** Main class of constituents present in the essential oils tested (relative abundance expressed in percentage).

Clade	Asterids					Magnoliids		Rosids									Mixture (1:1)				
Order	Lamiales				Poales	Laurales			Myrtales			Sapindales									
Family	Lamiaceae					Lauraceae			Myrtaceae				Rutaceae								
	Relative Percentage (%)																				
*Class of Compounds*	*L. h*	*M. p*	*O. b*	*A. t*	*C. c*	*C. z*	*L. c*	*P. g*	*E. g*	*M. a*	*S. a*	*B. s*	*C. a*	*C. b*	*C. l*	*C. r*	M1	M2	M3	M4	M5
**Monoterpene Hydrocarbons (MH)**	6.4	6.9	2.3	66.0	3.9	15.5	21.3		9.1	41.6		84.3	97.4	49.0	94.3	99.7	12.6	42.3	42.5	36.7	2.2
**Oxygenated Monoterpenes (OM)**	85.0	87.8	56.1	26.4	86.3	7.4	75.7	83.4	90.5	39.1		6.7	1.9	48.5	3.6		80.0	51.2	49.0	56.0	1.9
**Sesquiterpene Hydrocarbons (SH)**	5.4	4.6	20.0	4.7	4.5	14.7	0.9	7.8	0.3	14.8	9.5	3.7	0.2	2.4	2.0	0.1	3.4	2.9	4.3	3.5	10.1
**Oxygenated Sesquiterpenes (OS)**	1.3	0.3	7.9	1.9	0.9	0.8		6.9	0.1	1.8	0.4						0.5	1.2	1.2	0.8	1.2
**Phenylpropanoides (PP)**			12.7		2.0	60.3		1.2			90.1	0.2									64.4
**Non-terpenes (NT)**	1.9	0.2	0.2	0.5	2.0	1.0	1.5			0.9			0.5	0.1	0.1		1.7	0.9	1.2	1.7	20.1

Legend: *L. h*: *Lavandula hybrida*, *M. p*: *Mentha piperita*, *O. b*: *Ocimum basilicum*, *A. t*: *Aloysia triphilla*, *C. c*: *Cymbopogon citratus*, *C. z*: *Cinnamomum zeylanicum*, *L. c*: *Litsea cubeba*, *P. g*: *Pelargonium graveolens*, *E. g*: *Eucalyptus globulus*, *M. a*: *Melaleuca alternifolia*, *S. a*: *Syzygium aromaticum*, *B. s*: *Boswellia sacra*, *C. a*: *Citrus aurantium*, *C. b*: *Citrus bergamia*, *C. l*: *Citrus limon*, *C. r*: *Citrus reticulata*, M1: *Litsea cubeba–Cymbopogon citratus*, M2: *Aloysia triphylla–Listea cubeba*, M3: *Aloysia triphylla–Cymbopogon citratus*, M4: *Aloysia triphylla–Litsea cubeba–Cymbopogon citratus*, M5: *Cinnamomum zeylanicum–Syzygium aromaticum*; MH: Monoterpene Hydrocarbons, OM: Oxygenated Monoterpenes, SH: Sesquiterpene Hydrocarbons, OS: Oxygenated Sesquiterpenes, PP: Phenylpropanoides, NT: Non-Terpenes.

**Table 3:** Antimicrobial activity expressed as growth inhibition zone and minimum inhibitory concentration of the sixteen EOs and five mixtures against *Escherichia coli* and *Aspergillus fumigatus* strains.

Essential Oil	*Escherichia coli* MIC (mg/mL)	*Aspergillus fumigatus* MIC (mg/mL)
*Aloysia tryphilla*	ne	0.855
*Boswellia sacra*	ne	>8.50
*Cinnamomum zeylanicum*	2.52	5.05
*Citrus aurantium*	ne	>8.50
*Citrus bergamia*	ne	8.70
*Citrus limon*	ne	4.25
*Citrus reticulata*	ne	4.25
*Cymbopogon citratus*	1.118	0.895
*Eucalyptus globulus*	ne	4.575
*Lavandula hybrida*	ne	8.85
*Litsea cubeba*	1.106	1.770
*Ocimum basilicum*	9.15	9.15
*Melaleuca alternifolia*	ne	1.780
*Mentha piperita*	1.14	9.12
*Pelargonium graveolens*	17.8	>8.90
*Syzygium aromaticum*	1.318	8.95
M1	4.449	>17
M2	8.75	>17.5
M3	4.349	>17.4
M4	3.292	>26.35
M5	2.578	>20.65
Amoxycillin-clavulanic acid	0.008/0.004	
Voriconazole		1

Legend—MIC: minimum inhibitory concentration; M1: *Litsea cubeba–Cymbopogon citratus*, M2: *Aloysia triphylla–Listea cubeba*, M3: *Aloysia triphylla–Cymbopogon citratus*, M4: *Aloysia triphylla–Litsea cubeba–Cymbopogon citratus*, M5: *Cinnamomum zeylanicum–Syzygium aromaticum*; ne: no effective.
